# Melatonin protects uterus and oviduct exposed to nicotine in mice

**DOI:** 10.2478/intox-2014-0007

**Published:** 2014-07-16

**Authors:** Seyedeh Nazanin Seyed Saadat, Fahimeh Mohammadghasemi, Sina Khajeh Jahromi, Mohammad Amin Homafar, Mostafa Haghiri

**Affiliations:** 1 Student Research Committee Office, Guilan University of Medical, Sciences, Rasht, Iran; 2 Cellular & Molecular Research Center, Faculty of Medicine, Guilan University of Medical Sciences, Rasht, Iran; 3 Faculty of Medicine, Guilan University of Medical Sciences, Rasht, Iran

**Keywords:** nicotine, melatonin, uterus, oviduct, estrogen receptor

## Abstract

Smoking is associated with higher infertility risk. The aim of this study was to evaluate protective effects of melatonin on the uterus and oviduct in mice exposed to nicotine. Adult female mice (n=32) were divided into four groups. Group A: control animals received normal saline, Group B: injected with nicotine 40µg/kg, Group C: injected with melatonin 10 µg, Group D: injected with nicotine 40µg/kg and melatonin 10 µg. All animals were treated over 15 days intraperitoneally. On the 16th day, animals in the estrus phase were dissected and their uterus and oviducts were removed. Immunohistochemistry was recruited for studying apoptosis and for detection of estrogen receptor (ER) alpha in luminal epithelium of the uterus and oviduct. Enzyme-linked immunosorbent assay was used for serum estradiol level determination. Nicotine in group B decreased estradiol level and ERalpha numbers both in the uterus and oviduct (*p<*0.05). Co-administration of melatonin-nicotine in Group D ameliorated the histology of the uterus and oviduct, increased ERalpha numbers and reduced apoptosis in the uterus and oviduct compared with the nicotine Group B (*p<*0.05). This study indicates that nicotine impairs the histology of the uterus and oviduct and co-administration of melatonin-nicotine ameliorates these findings, partly through alteration in ERalpha numbers and reduction of apoptosis.

## Introduction

Cigarette smoking is associated with reproductive life impairment, such as earlier onset of menopause, higher infertility risk, lower fecundity rate and lower in vitro fertilization (IVF) success rate (Dechanet *et al.*, 2011).

Nicotine is considered one of the most important components of cigarette smoke. Several studies have reported effects of nicotine on the endometrium and myometrium (Dechanet *et al.*, 2011). Nicotine impairs fertility, reduces uterus weight, endometrial and myometrial thickness (Tuttle *et al.*, 2009) with a direct effect on the morphology of ovaries and estradiol levels in rats (Sanders *et al.*, 2002) or mice (Mohammadghasemi *et al.*, 2012). Fetal or neonatal exposure to nicotine was found to induce apoptosis in rat ovary granulosa cells (Petrik *et al.*, 2009). The morphology and functional integrity of the oviducts and uterus are evidently estrogen dependent. Estrogen plays key roles in the development and maintenance of normal sexual and reproductive functions (Heldring *et al.*, 2007). The biological effects of 17β-estradiol (E_2_) are mediated through activation of estrogen receptors (ERs), a ligand-dependent transcription factor belonging to the nuclear hormone receptor super-family (Shao *et al.*, 2012). In rodents and mammals, ERalpha is the predominant ER subtype in the fallopian tubes and uterus and plays a crucial role in physiological processes of the female reproductive tract (Wang *et al.*, 2000; Shao *et al.*, 2012). Thus alteration in expression of ERalpha may be associated with both function and morphology of the uterus and oviducts. Previous studies reported that both smoking (Sanders *et al.*, 2002; Dechanet *et al.*, 2011) and nicotine alone reduced the estradiol level (Petrik *et al.*, 2009).

It is well known that melatonin, a pineal hormone, plays a key role in regulating several reproductive processes (Tamura *et al.*, 2009). It is a powerful antioxidant and indirect free radical scavenger which can detoxify both reactive oxygen and reactive nitrogen species and stimulates antioxidant enzymes (Tamura *et al.*, 2009). Melatonin receptors have been detected in the female reproductive tract including granulose cells, corpus luteum and myometrium (Tamura *et al.*, 2009). Melatonin controls the growth of cells through modulation of estrogen and progesterone receptors and has a direct effect on the cell cycle, cell differentiation and gap junction (Abd-Allah *et al.*, 2003; Garcia-Navarro *et al.*, 2007). It regulates the expression of sex steroid receptors in female reproductive tissues (Chuffa *et al.*, 2013). It inhibits apoptosis (Ghasemi *et al.*, 2010) and affects steroidogenesis through melatonin MT1 and MT2 receptors in the female reproductive tract (Woo *et al.*, 2001). Under treatment with nicotine, exogenous melatonin was found to exert a protective function on the ovary (Mohammadghasemi *et al.*, 2012). Most of the previous studies evaluated the effect of cigarette smoking on female fertility, yet little is known about the effect of nicotine on the endometrium or oviduct**.**

The aim of this study was to investigate the protective effect of simultaneous administration of a low dose of 10µg melatonin on the uterus and oviduct in mice under chronic treatment with 40µg/kg nicotine, one of the most important ingredients of cigarettes.

## Materials and methods

### Animals and treatment

Female adult NMRI mice (35–45 g) were purchased from Razi Institute, Karaj-Iran. All animals were housed in groups of eight to ten in cages under standard lighting conditions and with free access to water and food at 25°C. The animals were maintained and handled according to the protocols approved by the Guilan University of Medical Sciences Animal Care and Use Committee. Initially, the animals were randomly divided into four groups. The first group: control animals treated with normal saline, the second group: animals were injected with nicotine 40µg/kg (Sigma, USA). The third group: animals were injected with melatonin (Sigma, USA), 10µg. The fourth group: animals were injected with nicotine 40µg/kg and melatonin 10µg. In all groups the animals were injected over 15 days. All injections were performed intraperitoneally.

Melatonin was dissolved in 95% ethanol and 0.9% NACL (1:7v/v) and was administered between 5.00–6.30 pm. After the melatonin and nicotine treatment period, all animals were monitored by vaginal smears. The estrous cycle was characterized by four phases: proestrus with numerous nucleated epithelial cells, some squamous epithelial cells and few leukocytes, estrus with many clusters of squamous epithelial cells, metestrus with some nucleated and squamous epithelial cells and abundant leukocytes, and diestrous with few cells and presence of thick mucus.

Animals in the estrus phase were chosen and placed in 4 groups, each comprising 8 mice. On day 16, all animals were dissected and their blood samples were collected. Their uterus (part nearer to the ovary) and oviducts were removed. Tissue samples were fixed in 10% neutral buffered formalin and were processed routinely for histological studies. Then 5-µm sections were prepared and stained with H&E or for immunohistochemical assessments to detect apoptosis and ERalpha luminal epithelial cells.

### Histologic study

In each animal 2–3 slides and in each slide 5 microscopic fields were observed using a light microscope (Olympus Japan) with a magnification of 400×. The numbers of endometrial glands in the uterus or mucosal folds in the oviduct were counted.

### Hormone measurement

Blood samples were collected through the inferior vena cava immediately after sacrificing the mice. The serum was separated and stored at –80°C. Estradiol levels were measured using an enzyme linked immunosorbent assay (ELISA) kit according to the manufacturer's instructions (Monobind,USA). Serum references (25µL) were dispensed into the wells. After adding the estradiol biotin reagent (50µL) into the wells, microplates were swirled, covered and incubated for 30 minutes at room temperature. In the next step, estradiol enzyme reagent (50µL) was added to each well and swirled, covered and incubated for 90 minutes at room temperature. After discarding the contents of the microplates, 350µL wash buffer was added and this was repeated three times. A dose of 100µL substrate solution was added to the wells and incubated at room temperature for 20 minutes. Finally, stop solution (50µL) was added to each well for stopping the enzymatic reaction and then the absorbance of each well was read at 450 nm.

### Detection of ERalpha

Sections were dewaxed and rehydrated. Deparaffinization was carried out in xylene and graded ethanols. Endogenous peroxidase was blocked by incubation in 5% H_2_O_2_ in methanol for 15 min. Heat-mediated antigen retrieval was performed by microwaving three times for 5 min at 500W in Citra-Plus solution (Biogenex; San Ramon, Ca) for ER staining. Unspecific bindings were blocked using the biotin blocking system (Dako; Carpinteria,Ca). Incubation was carried out with the mouse monoclonal antibody1D5, dilution 1:200 (Dako), 1h at room temperature. Sections were then counterstained with hematoxylin, dehydrated, and observed under a light microscope. Cells with a brown nucleus revealed the presence of ERalpha. Cells with a blue nucleus were considered negative for the presence of ERalpha. In each animal, 2 slides and in each slide 8–10 fields with an area of 1×1 mm^2^ were studied. In each field the number of ERalpha positive cells were counted and then divided by numbers of both positive and negative cells and expressed in percentages.

### Detection of apoptotic cells

*In situ* detection of cells with DNA strand breaks was performed in formalin-fixed, paraffin-embedded tissue sections by terminal deoxynucleotidyl transferase dUTP nick-end labeling (TUNEL) according to the manufacturer's instructions (Roche, Germany), as described previously (Ghasemi *et al.*, 2010). Cells with a dark-brown nucleolus were considered apoptotic. In each animal 2 slides and in each slide 8–10 fields with an area of 1×1mm^2^ were studied. In each field the number of positive apoptotic cells was counted and then divided by numbers of both positive and negative cells and expressed in percentages.

### Statistical analysis

Data analyses were performed using SPSS version 13.0 for windows microsoft year 2010. The normality distribution of samples was tested using the Kolmogorov-Smirnov test. Then the data were analyzed by analysis of variance (ANOVA) and Tukey *post-hoc* tests. The value *p<*0.05 was considered evidence for statistical significance.

## Results

### Histological assessment

**Figure 1:**
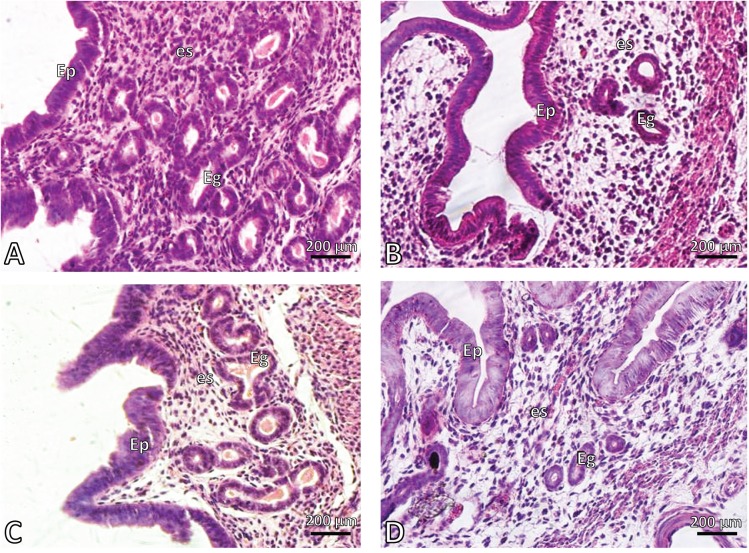
Photomicrograph of mouse endometrium. **A:** control, **B:** nicotine, **C:** melatonin and **D:** melatonin+nicotine treated mice. EP: epithelium, es: estroma, Eg: endometrial glands. H&E. Magnification 400×.

**Figure 2:**
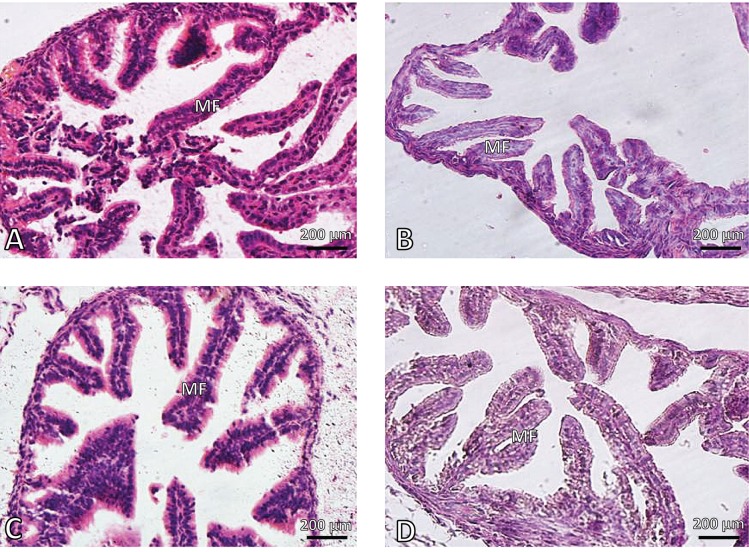
Photomicrograph of mouse oviduct. **A:** control, **B:** nicotine, **C:** melatonin and **D:** melatonin+nicotine treated mice. Mf: mucosal folds. H&E. Magnification 400×.

Nicotine induced morphological alterations in both the uterus endometrium and oviduct mucosa. The mean number of endometrial glands in the nicotine treated group was 6.4±6.2 vs 14.2±4.1 in the controls (*p<*0.05). Nicotine reduced significantly both endometrial glands and mucosal folds of the oviducts compared with the controls (*p<*0.05). Co-administration of nicotine-melatonin significantly increased the number of endometrial glands compared with the nicotine group (*p<*0.05) (Figures 1 – 2 and Table 1). The number of mucosal folds in the oviducts of the co-administered group was increased compared with the nicotine group, yet this value was not significant (Table 1).

**Table 1:** Effect of nicotine and melatonin on adult mouse uterus and oviduct.

O. folds Numbers/field	E. glands Numbers/field	Eralpha oviduct/field	ERalpha uterus/field	Estradiol level (pg/ml)	Groups
18.2±3.4	14.2±4.1	11.00±2.20^b^	15.25±1.46^b^	57.25±4.43^b^	Control
10.8±2.4^a^	6.4±6.2^a^^b^	4.25±1.03^a^	10.56±1.63^a^	41.37±3.70^a^	Nicotine
16.3±3.1	12.1±3.0^b^	13.00±2.44^b^	12.25±1.05^b^	53.25±5.14^b^	Melatonin
15.2±4.0	10.6±2.8^b^	7.66±2.53^a^^b^	13.85±0.64^b^	50.62±3.96^a^^b^	Nicotine+melatonin

Data are expressed as Mean±S.D.

a significant from controls *p<*0.05

b significant from nicotine group *p*<0.05; E: Endometrial; O: Oviduct

### Serum estradiol (E2) level and detection of ERalpha

**Figure 3:**
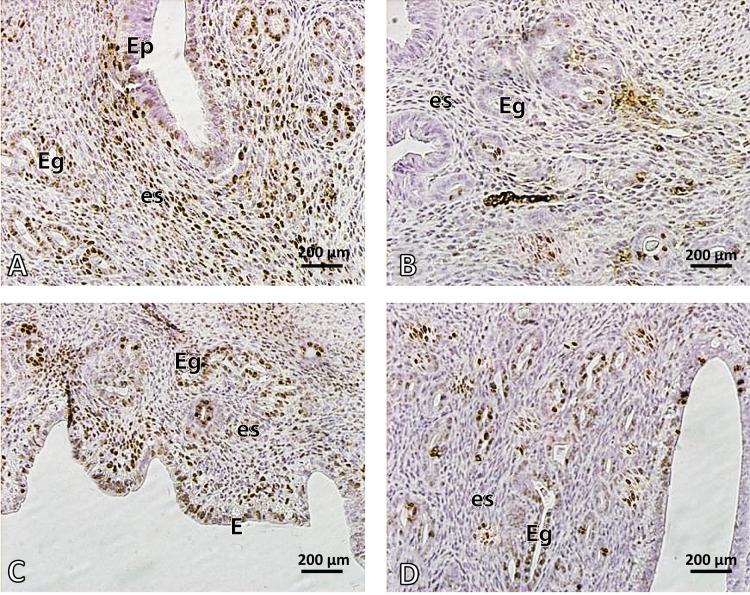
Photomicrograph of mouse endometrium. **A:** control, **B:** nicotine, **C:** melatonin and **D:** melatonin+nicotine treated mice. Brown cells show ER alpha positive cells. Arrows show endometrial glands. Note the reduced numbers of ER alpha positive cells and endometrial glands in nicotine (**B**) compared with control (**A**). In **D**, melatonin ameliorated the endometrium and increased ERα. EP: epithelium, es: estroma, Eg: endometrial glands. Magnification 400×.

**Figure 4:**
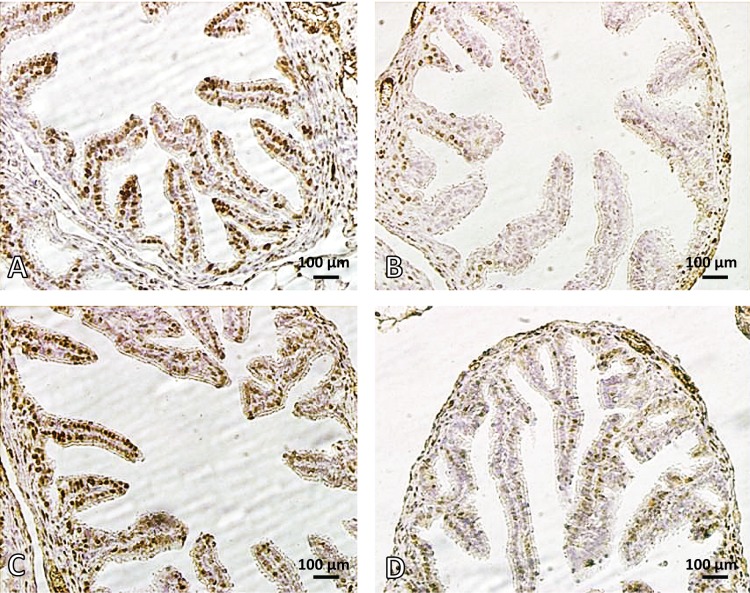
Photomicrograph of mouse ampulla (oviduct). **A:** control, **B:** nicotine treated mouse, **C:** melatonin treated mouse and **D:** melatonin+nicotine treated mouse. Brown cells show ER alpha positive cells. Note the reduced numbers of ER alpha positive cells and mucosal folds (MF) in nicotine (**B**) compared with control (**A**). In **D**, melatonin ameliorated the mucosal folds of the oviduct and increased ER alpha. ER alpha Immunostaining. Magnification 400×.

Administration of nicotine in the dose of 40µg/kg for 15 days reduced significantly the serum estradiol (E2) level and ERalpha in epithelial cells of the uterus and oviducts compared with the controls (*p<*0.05). Melatonin alone did not affect these parameters. However, co-administration of melatonin and nicotine increased significantly the serum estradiol (E2) level and ER alpha in epithelial cells of the uterus and oviducts compared with the nicotine group (*p<*0.05) (Table 1, Figures 3 – 4).

### Detection of apoptosis

**Figure 5:**
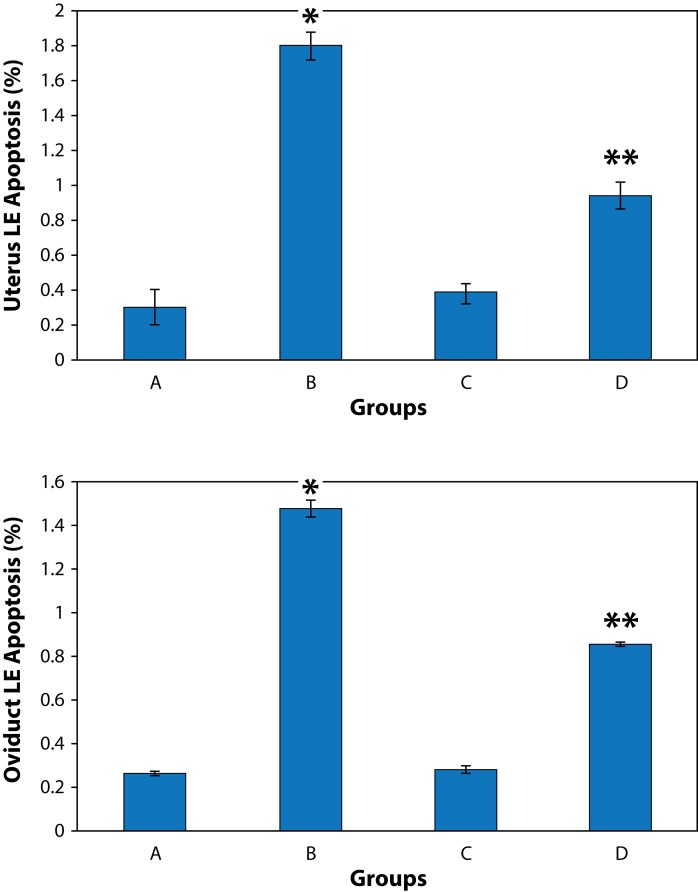
Effect of nicotine and melatonin on apoptosis in luminal epithelium (LE) in mouse uterus and oviduct. **A:** control, **B:** nicotine, **C:** melatonin, **D:** melatonin+nicotine treated groups. Data are expressed as Mean± SD. * shows significant data from controls (A), *p<*0.05. ** shows significant data from both controls (**A**) and nicotine alone treated group (**B**), *p<*0.05.

A few apoptotic cells were scattered in the luminal or glandular epithelium of the oviducts and uterus in controls. The percentage of apoptotic cells was significantly increased both in the uterus and oviducts in the nicotine treated group compared to controls (*p<*0.05). The nicotine-melatonin combination significantly reduced apoptosis in epithelial cells of the uterus and oviducts compared with the nicotine alone group (*p<*0.05) (Figure 5).

## Discussion

In this study, melatonin was found to exert protective effects on the uterus and oviduct in mice under treatment with nicotine. Nicotine administration was shown to be associated with a reduction in estradiol level and ERalpha numbers and it increased apoptosis both in the uterus and oviduct in mice.

Apoptosis is programmed physiological cell death that occurs in all types of cells in both embryonic and adult periods (Petrik *et al.*, 2009). Due to the cyclic nature of the female reproductive system, the ovary, the endometrium and the mammary gland sustain continuous cycles of cell growth and apoptosis in response to hormonal changes (Meresman, 2011). Apoptotic mechanisms exploited by nicotine include alteration of the bcl_2_/bax ratio and activation of the caspase-3 pathway (Petrik *et al.*, 2009). In this study, nicotine was found to induce an increase in the number of apoptotic cells in the epithelium of the uterus and oviduct. The increased level of apoptosis in the luminal epithelium of the uterus and oviduct is presumably due to a reduced level of estradiol and its receptor, as reported previously (Petrik *et al.*, 2009; Wang *et al.*, 2000). Our study showed that co-administration of melatonin-nicotine reduced apoptotic cells in luminal epithelial cells in the uterus and oviducts. Similarly, an antiapoptotic effect of melatonin was demonstrated in different tissues (Guneli *et al.*, 2008; Take *et al.*, 2009). The mechanisms related to antiapoptotic effects of melatonin include: bcl_2_ expression by mitochondrial pathways and reduction of caspase-3 activity (Tamura *et al.*, 2009), antioxidative effect of melatonin, alteration in cell differentiation, growth factors, cell division and cell attachments (Garcia-Navarro *et al.*, 2007), which were however not evaluated in the present study. A further study with focus on molecular mechanisms of apoptosis and interaction between melatonin receptors, estrogen receptors and nicotine acetylcholine receptors following treatment with nicotine and melatonin is therefore suggested.

Our study showed that nicotine reduced estradiol levels. Estrogen is primarily produced by preovulatory follicles under the influence of FSH in the rodent ovary (Byers *et al.*, 1997; Chuffa *et al.*, 2013). Estrogen has various intra-ovarian roles during follicogenesis. In granulosa cells of maturing follicles, estradiol has little effect alone but is required for maximum FSH stimulation of estradiol synthesis, LH receptor expression, LH responsiveness, antrum formation, gap junction formation, and prevention of atresia (Emmen *et al.*, 2005). Smokers show abnormal endocrine profiles of testosterone, FSH, LH and estradiol levels (Dechanet *et al.*, 2011). Epidemiologic studies reported that women who smoke had lower serum estrogen levels than nonsmokers (Barbieri *et al.*, 2005). Increased estrogen hepatic metabolism and decreased estrogen synthesis are the probable mechanisms for the antiestrogenic effect of cigarette smoking (Michnovicz *et al.*, 1986). In this study, the decreased estrogen level in the nicotine group can probably be explained by its toxic effects on the ovary or granulosa cell function (Mohammadghasemi *et al.*, 2012). However, Sanders *et al.* (2002) did not find any changes in estradiol levels of the bovine theca interna and granulose cells following nicotine administration. In contrast, Gocze *et al.* (1997) and Bodies *et al.* (1997) observed a slight increase in estradiol production by human granulose cells treated with nicotine. One reason for these conflicting results may be due to species differences. Further reasons could include the source of cells and the length of culture period, variation in the form of the nicotine preparations used, as well as nicotine dosage and duration of nicotine exposure (Sanders *et al.*, 2002).

In our study, ERalpha was reduced in the epithelial tissue of both the uterus and oviduct in nicotine treated animals. Steroid hormone action is mediated via nuclear receptor proteins ERalpha and ERbeta, which function as ligand modulated transcription factors. In mammals and rodents, the activity of ERalpha is regulated by cycling hormone levels (Wang *et al.*, 2000). E2 was found to induce increased concentrations of both ER alpha and progesterone receptor (Wang *et al.*, 2000). The lowered level of estradiol in this study reduced presumably the numbers of ERalpha. E2 was shown to enhance ER alpha mRNA synthesis in the rat uterus 24 h after injection and the uterine responses to E2 and progesterone are directly or indirectly mediated by the cell specific expression of their receptors (Wang *et al.*, 1999).

Our study showed that co-administration of melatonin-nicotine increased both the estradiol level and ER alpha, compared with the group that received nicotine alone. Melatonin has specific receptors MT1 and MT2 both in the uterus and oviduct (Tamura *et al.*, 2009). Depending on the tissue, organ and species, melatonin activates different second messenger cascades by interacting with the same receptor subtype (Tamura *et al.*, 2009). Such alteration in the co-administered group may be due either to the direct effect of melatonin on both uterine and oviduct tissue or to the effect of melatonin on the hypothalamo-pituitary-gonadal axis (HPG). Melatonin interacts with estrogen-signaling pathways through indirect neuroendocrine mechanisms, direct actions at the tumor cell level, and regulation of enzymes involved in the biosynthesis of estrogens (Yoo & Jeung, 2009). It influences sex steroid production at different stages of ovarian follicular maturation (Adriaens *et al.*, 2006). There are controversial results about the effect of melatonin on sex hormones. Melatonin treatment increases mRNA expression of LH but not FSH receptors in human granulose cells (Tamura *et al.*, 2009). Melatonin increases the production of progesterone and androgen in porcine antral follicles, without effect on the estradiol level (Tanavde & Maitra 2003). However in hamsters, it increases ER activity in the uterus (Danforth *et al.*, 1983) and decreases the production of progesterone and estradiol (Tamura *et al.*, 2009). Abd-Allah *et al.* (2003) and Chuffa *et al.* (2013) found reduced numbers of ER in the rat uterus after administration of melatonin. Similarly, melatonin increasesd calbindin families of calcium-binding proteins expression through ERalpha. These conflicts are due to the cell type, dosage and duration of melatonin treatment, experimental model, and species (Tamura *et al.*, 2009).

Our study showed co-administration of nicotine-melatonin reduced apoptosis and increased ER alpha compared to nicotine alone. These alterations probably suggest a functional link between increased cell survival and the up-regulation of ERalpha by melatonin induced ERalpha during nicotine-mediated cell death. In this regard, it has been shown that melatonin increases survival of rat pituitary GH3 cells against cell death mediated by H_2_O_2_ through ERalpha (Yoo & Jeung, 2009). Estrogen receptor alpha in other tissues also protects podocytes against apoptosis in vitro or in vivo (Kummer *et al.*, 2011).

In conclusion, this study indicates that melatonin exerts a favorable effect on the uterus and oviduct in mice treated with nicotine through reduction of apoptosis and modification in ERalpha in the luminal epithelium of the uterus and oviduct. Our results also suggest that melatonin may have a significant beneficial effect for clinical applications and subfertility or infertility induced by smoking or nicotine replacement therapy in women. Future studies on the current topic in humans are therefore recommended.
